# Epigenetic profiling of prostate cancer reveals potential prognostic signatures

**DOI:** 10.1007/s00432-024-05921-0

**Published:** 2024-08-24

**Authors:** Simon Bernatz, Ian G. Reddin, Tim R. Fenton, Thomas J. Vogl, Peter J. Wild, Jens Köllermann, Philipp Mandel, Mike Wenzel, Benedikt Hoeh, Scherwin Mahmoudi, Vitali Koch, Leon D. Grünewald, Renate Hammerstingl, Claudia Döring, Patrick N. Harter, Katharina J. Weber

**Affiliations:** 1 Goethe University Frankfurt, University Hospital, Clinic for Radiology and Nuclear Medicine, Frankfurt am Main, Germany; 2 Goethe University Frankfurt, University Hospital, Dr. Senckenberg Institute for Pathology, Frankfurt am Main, Germany; 3 Frankfurt Cancer Institute (FCI), Frankfurt am Main, Germany; 4 School of Cancer Sciences, University of Southampton, Southampton, UK; 5 Frankfurt Institute for Advanced Studies (FIAS), Frankfurt am Main, Germany; 6 Goethe University Frankfurt, University Hospital, Department of Urology, Frankfurt am Main, Germany; 7 Goethe University Frankfurt, University Hospital, Neurological Institute (Edinger Institute), Frankfurt am Main, Germany; 8 German Cancer Consortium (DKTK) Partner Site Frankfurt/Mainz, Frankfurt am Main, Germany; 9 German Cancer Research Center (DKFZ), Heidelberg, Germany; 10 Goethe University Frankfurt, University Hospital, University Cancer Center (UCT), Frankfurt am Main, Germany; 11 Center for Neuropathology and Prion Research, Faculty of Medicine, LMU Munich, Munich, Germany; 12 German Cancer Consortium (DKTK), Partner Site Munich, A Partnership Between DKFZ and University/University Hospital, LMU Munich, Munich, Germany

**Keywords:** Prostate cancer, Radiomics, DNA methylation-based tumor deconvolution

## Abstract

**Purpose:**

While epigenetic profiling discovered biomarkers in several tumor entities, its application in prostate cancer is still limited. We explored DNA methylation-based deconvolution of benign and malignant prostate tissue for biomarker discovery and the potential of radiomics as a non-invasive surrogate.

**Methods:**

We retrospectively included 30 patients (63 [58–79] years) with prostate cancer (PCa) who had a multiparametric MRI of the prostate before radical prostatectomy between 2014 and 2019. The control group comprised four patients with benign prostate tissue adjacent to the PCa lesions and four patients with benign prostatic hyperplasia. Tissue punches of all lesions were obtained. DNA methylation analysis and reference-free *in silico* deconvolution were conducted to retrieve Latent Methylation Components (LCMs). LCM-based clustering was analyzed for cellular composition and correlated with clinical disease parameters. Additionally, PCa and adjacent benign lesions were analyzed using radiomics to predict the epigenetic signatures non-invasively.

**Results:**

LCMs identified two clusters with potential prognostic impact. Cluster one was associated with malignant prostate tissue (*p* < 0.001) and reduced immune-cell-related signatures (*p* = 0.004) of CD19 and CD4 cells. Cluster one comprised exclusively malignant prostate tissue enriched for significant prostate cancer and advanced tumor stages (*p* < 0.03 for both). No radiomics model could non-invasively predict the epigenetic clusters.

**Conclusion:**

Epigenetic clusters were associated with prognostically and clinically relevant metrics in prostate cancer. Further, immune cell-related signatures differed significantly between prognostically favorable and unfavorable clusters. Further research is necessary to explore potential diagnostic and therapeutic implications.

**Supplementary Information:**

The online version contains supplementary material available at 10.1007/s00432-024-05921-0.

## Introduction

Prostate cancer (PCa) is the second most common cancer in men (Sung et al. 2021). Advances in multiparametric MRI (mpMRI) improved patient management and biopsy techniques (Mottet et al. 2019). Yet, tumor heterogeneity can compromise pathologic confirmation of diagnosis (Stewart et al. 2013; Guo et al. 2018). The comprehensive molecular characterization of PCa is the basis for effective biomarker development (Guo et al. 2018). However, biopsies not only sample tumor cells but also the adjacent tumor microenvironment, which impacts genomic analysis and interpretation of results (Aran et al. 2015). Here, epigenetic analyses can be a way forward. For example, leucocytes unmethylation for purity (LUMP) measures the immune counterparts in a tissue sample by averaging 44 non-methylated immune-specific CpG sites to assign an immune cell estimate (Aran et al. 2015). Such immune cell signatures might reflect clinically relevant tumor characteristics (Aran et al. 2015). Furthermore, radiomics describes the transformation of medical images into mineable data to leverage artificial intelligence to non-invasively characterize the whole tumor without sampling bias, and it has shown promising results in describing tumor phenotypes beyond visual perception with prognostic impact (Bonekamp et al. 2018; Varghese et al. 2019).

We hypothesize that epigenetic signatures are associated with clinically relevant measures, such as malignancy and tumor stage. Further, we hypothesize that radiomics can serve as non-invasive surrogate for prognostically relevant epigenetic clusters.

## Materials and methods

The institutional Review Board of the Ethical Committee approved this retrospective study (project number: 20–890, Goethe University Frankfurt am Main, Germany).

### Study design

**Fig. 1:**
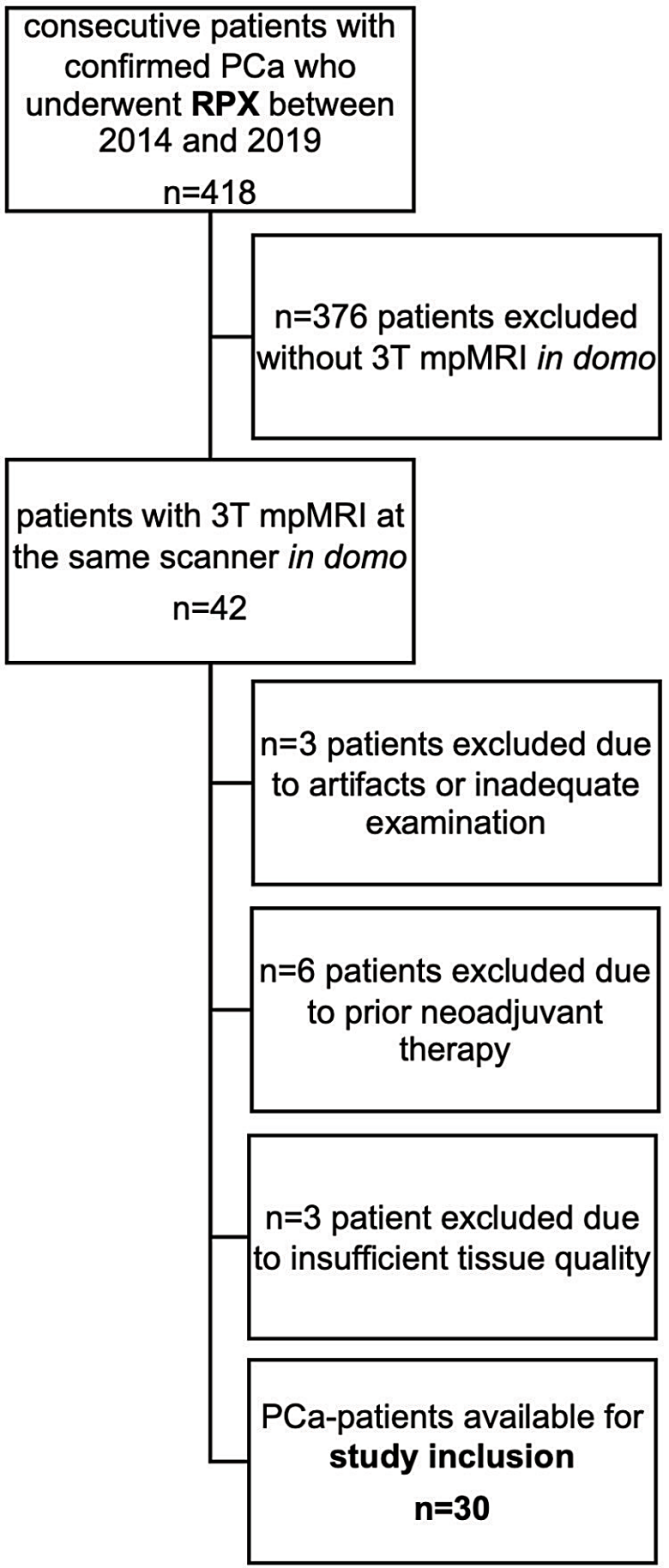
STARD flowchart of prostate cancer patient inclusion into the study. The flowchart depicts the retrospective inclusion of the 30 prostate cancer patients as previously described (Bernatz et al. 2020). Four additional retrospective patients with BPH (median age 70 [61–76]) served as complete benign control patients which were consecutively enrolled in clinical routine

Our study is an in-depth subgroup analysis of a previously reported patient cohort (Bernatz et al. 2020) with added novelty by epigenetic analysis, inclusion of a new control cohort with benign prostatic hyperplasia (BPH), and correlation with radiomics analysis. In short, 418 consecutive patients with confirmed PCa who had a mpMRI before radical prostatectomy (RPX) between 2014 and 2019 were screened for study inclusion to finally include a total of 30 patients (in comparison to the prior study (Bernatz et al. 2020) we had to exclude three patients with insufficient tissue quality for epigenetic analysis, therefore, resulting in 30 PCa patients). The further inclusion and exclusion criteria for the PCa patients are depicted in Bernatz et al. (2020). See Fig. 1 for the flow-chart of PCa-patient inclusion. Control patients were treated with holmium laser enucleation of the prostate (HoLEP) for BPH in 2019 and four patients were consecutively enrolled. The inclusion criteria for the control patients were (I) BPH, (II) no malignancy in pathologic analysis. Control exclusion criteria were (I) incidental malignancy in postoperative tissue specimens, (II) insufficient tissue quality. From four PCa patients, additional adjacent morphologically benign tissue was sampled for epigenetic analysis.

### Reference standard

All tissue samples were histologically confirmed in the institution’s pathology department by a uropathologist (JK). All PCa and adjacent benign tissue samples were correlated with the matching localization in the mpMRI as previously described (Bernatz et al. 2020).

### DNA methylation analysis and tumor deconvolution

The tissue samples were subjected to DNA methylation analysis using the Human Methylation EPIC array by Illumina (Illumina, California, USA). Formalin-fixed, Paraffin-embedded tissue was cut in 4 μm thin section with a microtome (Leica SM 2000R, Wetzlar, Germany), mounted on slides (Superfrost Plus, Thermo Scientific, Braunschweig, Germany) and H&E stained. Representative sections of the lesions were selected, and punch biopsies (1.0 mm diameter, kai Europe GmbH, Solingen, Germany) were taken for DNA isolation by use of the Stratek Invisorb Genomic DNA Kit II (stratek molecular, Berlin, Germany). After assessment of DNA concentration using the Qubit DNA BR Assay Kit and Qubit 3 Fluorometer device (Invitrogen, Life Technologies Corporation, Oregon, USA), DNA was further processed and hybridized to the Human Methylation EPIC array beadchips (Illumina, California, USA) following standard protocols provided by the manufacturer. EPIC array beadchips were scanned by an iScan (Illumina, California, USA) and raw intensity data (idats) was obtained. Idats were imported into the R software package “RnBeads” (Müller et al. 2019) to perform quality control, exploratory and differential methylation analysis as well as to obtain LUMP estimates. The LUMP algorithm uses measurements of leucocyte unmethylation to infer leukocyte infiltration in bulk tissue samples by the analysis of 44 CpG sites which are unmethylated in leukocytes and methylated in tumor cells (Aran et al. 2015). DNA methylation data was normalized using the “dasen” method from the R package “watermelon”.

Reference-free deconvolution of prostate tissue was performed using MeDeCom, which uses non-negative matrix factorization to compute Latent Methylation Components (LMCs; Scherer et al. 2020). LMCs represent methylation patterns shared between the samples’ most variable CpG sites - i.e. the top 5000 most variable CpG sites across all samples of this study - with correction for methylation patterns driven by patient age. LMCs are selected by evaluating cross-validation errors for LMCs numbers (kappa) and the regularization parameter (lambda). For each sample, proportions of LMCs were computed and subjected to hierarchical cluster analysis by use of Ward’s minimum variance method. LMCs-based clusters were further correlated with clinical tumor parameters and their cellular composition.

For reference-based deconvolution of prostate tissue we used MethylCIBERSORT as described in (Chakravarthy et al. 2018). In brief, idats are loaded into R, assessed for quality, Noob normalized and beta value calculated by use of the minfi package. An in silico cellular mixture matrix is generated by combining signature CpGs of immune cells (T regulatory cells, CD4 + effector cells, CD8 + T cells, CD20 + B cells, CD14 positive monocytes, eosinophils, neutrophils, NK cells), fibroblasts, endothelia and cancer cells with the samples’ CpGs to infer the estimates of cellular fractions present in the prostate tissue. Deconvolution of the files was realized on the CIBERSORT X platform provided by the Alizadeh and Newman labs (Newman et al. 2015).

### MRI imaging and examination

All imaging was performed on a single 3-T scanner and read in clinical routine as previously described (Bernatz et al. 2020), following the European Society of Urogenital Radiology (ESUR) guidelines. For the radiomics analysis, the MR images (T2-weighted (T2w), apparent diffusion coefficient (ADC), dynamic contrast-enhanced (DCE) were exported in “Digital Imaging and Communications in Medicine” (DICOM) format. Representative images of mpMRI acquisition are depicted in (Bernatz et al. 2020) and acquisition parameters are depicted in Supplementary Table 1.

### MRI segmentation

We depict the workflow of MRI segmentation in detail elsewhere (Bernatz et al. 2020). In short, we used the open-source 3D slicer computing platform (http://slicer.org, version 4.9.0) (Fedorov et al. 2012; Velazquez et al. 2013) to visualize and segment the whole 3-dimensional tumor volume of interest (VOI) of each tumor index lesion using ADC maps. Manual seeds were defined in each PCa index lesion with semi-automatic 3D-VOI annotation by grow-from-seeds algorithm (Velazquez et al. 2013; van Griethuysen et al. 2017). The benign adjacent tissue was manually defined. We depict representative images of the whole habitat index PCa lesion segmentation in Supplementary Fig. 1.

### Feature extraction

Within the 3D Slicer software platform, we used the open-source extension PyRadiomics (Pedregosa et al. 2011; Velazquez et al. 2013) to extract 105 radiomics features of seven feature classes as previously described (Bernatz et al. 2020).

### Quantitative radiographic biomarkers to predict epigenetic signatures

The analysis included 30 PCa patients with matching pathologic and radiologic index lesions. The control (BPH) patients did not have a mpMRI and were excluded from the radiomics machine learning analysis. All analyses were performed in Python 3.9.16. We used Pearson correlation analysis to drop all highly correlated (*r* > 0.95) features (*n* = 70) to reduce the risk of overfitting and to stratify our final radiomic features set. We split our dataset into an independent training (70%) and testing set (30%) with patient samples drawn at random. We scaled the features using StandardScaler (Bernatz et al. 2023) to have a mean value of 0 and a variance of ± 1. Next, we independently applied a pool of four variant machine learning models to predict the epigenetic signature clusters. We used different established machine learning models (I) logistic regression (LR), (II) random forest (RF), (III) ada boost (ADB) and (IV) stochastic gradient boosting (SGB). The machine learning pipeline is described in detail elsewhere (Virtanen et al. 2020). For each model, we depict the receiver operating characteristics (ROC) area under the curve (AUC) as implemented in scikit-learn 1.0.2 (Pedregosa et al. 2011).

### General statistical analysis

Statistical analyses were performed in JMP (JMP Statistical Software, SAS Institute, Cary, North Carolina, USA), R (R Core Team 2021), and Python, using SciPy (SciPy.stats) (Virtanen et al. 2020) and scikit-learn (Pedregosa et al. 2011) for further statistical analyses. Graphical illustrations were performed in Affinity Designer 2.1 (Serif (Europe) Ltd). The PCa sample size resulted from including all eligible patients according to the inclusion and exclusion criteria (Bernatz et al. 2020).

## Results

### Study population

Our study population comprised 34 tissue samples, including PCa (*n* = 30), benign tissue adjacent to PCa (*n* = 4), and non-malignant BPH (*n* = 4) of a total of 34 male patients (age PCa, 63 [58–79]; age BPH, 70 [61–76]). The adjacent benign tissue was sampled from four patients of the PCa cohort. PCa patients were treated with RPX. BPH patients were treated with HoLEP. The patient cohort is a subgroup of a previously published analysis (Bernatz et al. 2020). We depict the patient characteristics in Table 1 and the flow-chart of patient inclusion in Fig. 1.

**Table 1:** Clinical and epidemiological characteristics of included PCa patients

Variable	Study cohort
RPX	30 (100)
Median age at definite diagnosis (y)*	63 (58–79)
Median time (m)*, MRI to tissue (biopsy, RPX)	0 (0–7)
Mean PSA (ng/mL)**	11.81 (14.89)
Localization (index lesion)	
PZ	27
PZ/ AFS	3
PI-RADS, index lesion ***	
3	3 (10)
4	7 (23)
5	20 (67)
Gleason score, index lesion ***	
3 + 3	1 (3)
3 + 4	9 (30)
4 + 3	10 (33)
4 + 4	2 (7)
4 + 5	7 (23)
5 + 4	1 (3)

If not depicted otherwise, the numbers without parenthesis depict absolute numbers. * Data in round parenthesis are the min/max values; ** Data in round parenthesis is standard deviation; *** Data in round parenthesis are relative values; note: due to mathematical rounding, the summed relative values may differ slightly from 100. *AFS* anterior fibromuscular stroma; *m* months; *MRI* magnetic resonance imaging; *PI-RADS* Prostate Imaging Reporting and Data System; *PSA* prostate-specific antigen; *PZ* peripheral zone; *RPX* radical prostatectomy; *y* years

### Epigenetic signatures revealed two distinct LMC-based clusters

**Fig. 2:**
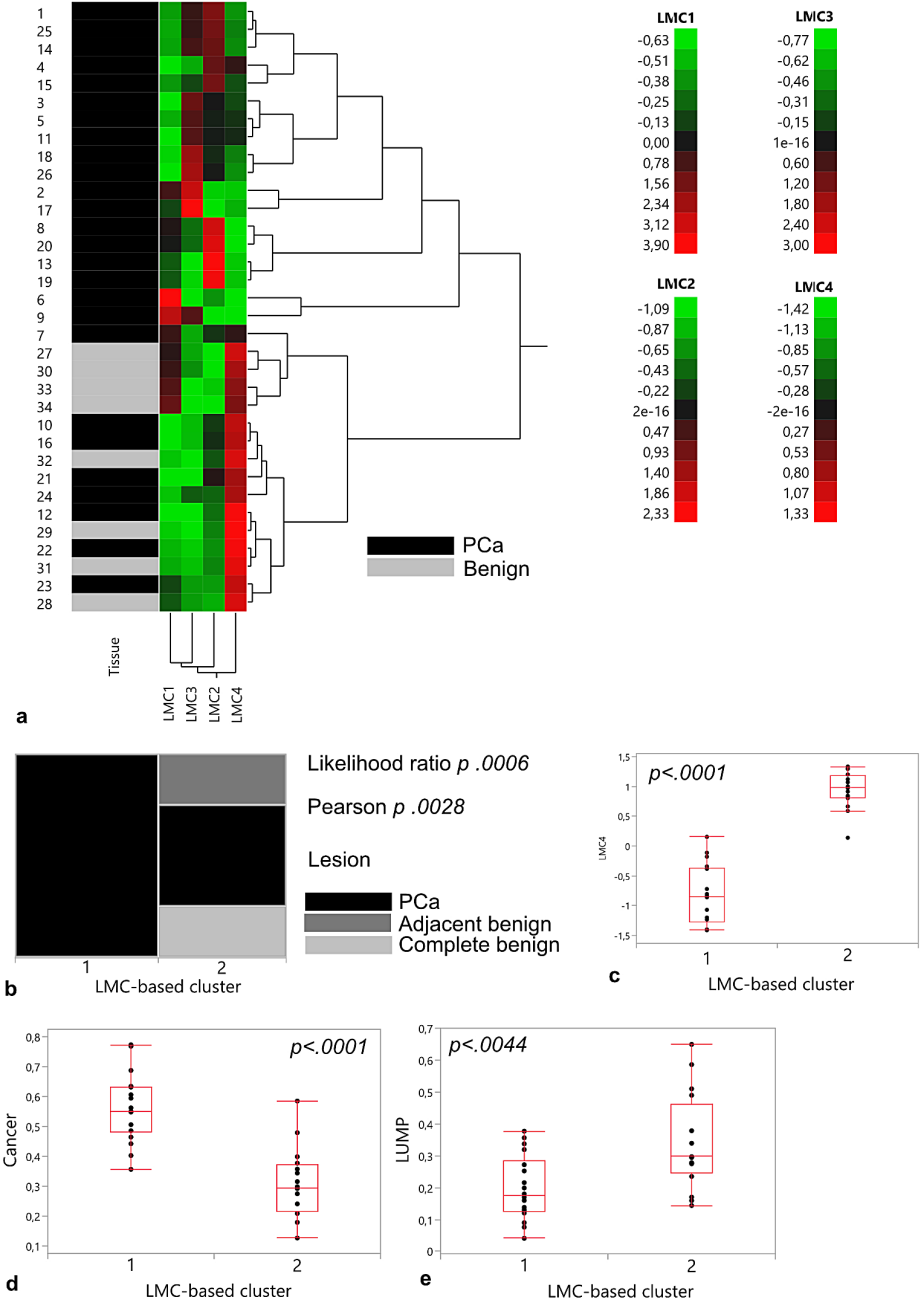
Latent Methylation Component-based unsupervised hierarchical clustering shows two clusters separating samples mainly according to malignancy status and MethylCIBERSORT-derived cancer cell estimates. **a** Hierarchical Clustering, Ward Method, LMC standardized, kappa = 4, lambda = 0.001; **b** Contingency table, p Chi2 test; **c** Distribution of LMC4 values within LMC-based clusters; **d** Cancer cell estimates, MethylCIBERSORT; **e** LUMP estimates; p Chi2 test. PCa Prostate Cancer

Large-scale DNA methylation profiles of prostate tissue were subjected to the reference-free deconvolution pipeline MeDeCom to compute major methylation patterns (Scherer et al. 2020). PCa samples of patients with adjacent benign tissue were excluded to avoid patients being represented twice in the data set. MeDeCom analysis rendered four LMCs, leading to two LMC-based clusters after unsupervised hierarchical cluster analysis (Fig. 2a). While LMC-based cluster 1 was composed of prostate cancer exclusively, LMC-based cluster 2 contained all benign and adjacent benign samples in addition to eight cancer samples (Fig. 2b). LMC 4 values predominantly discriminated between cluster allocation with higher LMC4 values indicative of cluster 2 (Fig. 2c). Reference-based deconvolution of LMC-based clusters showed cluster 1 to be composed of higher numbers of cancer cells (*p* < 0.0001) and lower numbers of leukocytes (LUMP, *p* = 0.0044) (Fig. 2d, e).

### Prostate tissue adjacent to tumor resembles complete benign controls on the epigenetic level

Next, we analyzed large-scale DNA methylomes to characterize the tissue samples adjacent to cancer lesions. Compared to PCa samples on a global DNA methylation level, two adjacent benign samples clustered rather with and two separately from cancer tissue (Supplementary Fig. 2a). We found 92 CpG sites within CpG islands, 17 within genes overall, and 10 within promoter regions being differentially methylated between adjacent benign and PCa (Table 2). Two differentially methylated sites on the promoter regional level were associated with the gene *ARHGAP42P1* (Rho GTPase Activating Protein 42 Pseudogene 1) (Supplementary Table 2). For the gene RPL35AP31 (Ribosomal Protein L35a Pseudogene 31), we found one CpG site each on a promoter and gene level to be significantly differentially methylated between PCa and adjacent benign tissue (Supplementary Tables 2, 3). Two adjacent benign samples clustered together with complete benign controls, two rather separated from controls in principal component analysis of global methylomes (Supplementary Fig. 2b). Of note, no CpG site was significantly differentially methylated between adjacent and complete benign tissue after correction for multiple comparisons (Table 2).

**Table 2:** Differentially methylated CpG sites

Differentially methylated CpG sites, genomic region	Adjacent benign vs. PCa	Adjacent benign vs. Complete benign
Genes	17	0
Promoters	10	0
CpG islands	92	0

Differentially methylated CpG sites according to genomic region in adjacent benign samples (*n* = 4) vs. PCa (*n* = 26) and complete benign tissue (*n* = 4), respectively. FDR adj. p values < 0.05

### Epigenetic clusters were associated with differential microenvironmental composition and prognostically relevant features

**Fig. 3:**
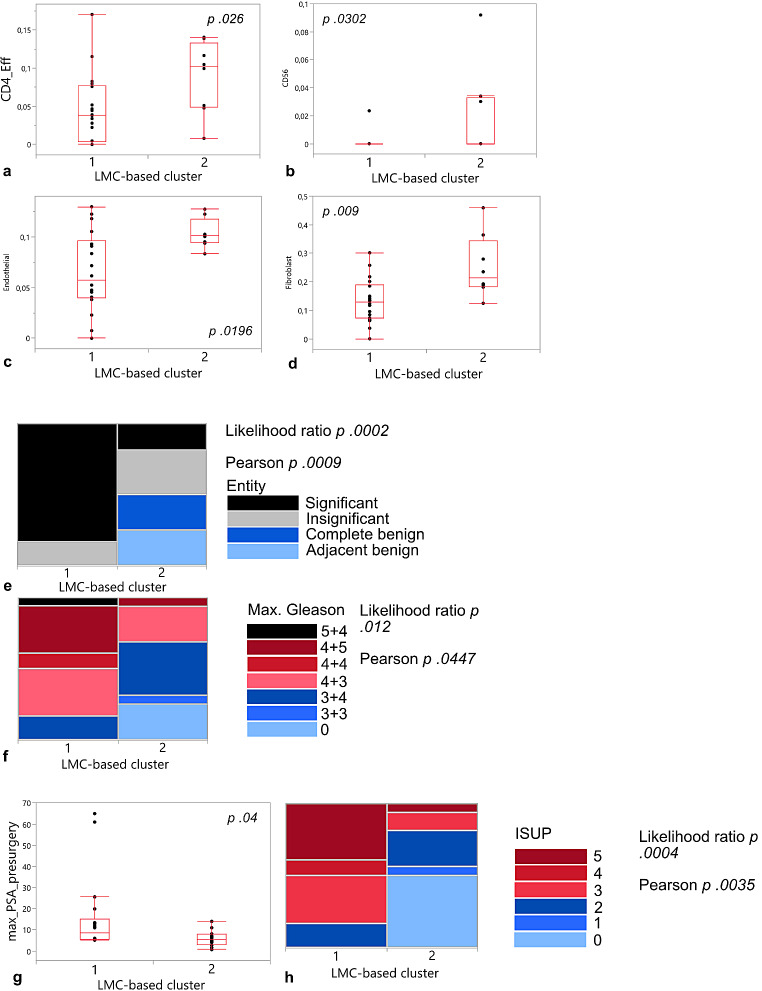
Cellular composition of prostate cancer samples with regard to allocation to LMC-based clusters with benign samples excluded from cluster 2, and association of LMC-based clusters with clinical parameters. MethylCIBERSORT-based tumor deconvolution for **a** CD4 effector cells; **b** CD56 + NK cells; **c** endothelial cells; **d** fibroblasts. P Chi square test. LMC-based cluster association with **e** Entitiy; **f** Maximal Gleason Score; **g** Maximal PSA presurgery; **h** ISUP

Next, we aimed to delineate differences in microenvironmental composition between the PCa clusters (benign cases were excluded for this analysis) by conducting the reference-based tumor deconvolution algorithm MethylCIBERSORT. Cluster 2 held samples with higher proportions of CD4 + effector cells (*p 0.026*), CD56 + NK cells (*p 0.0302*), endothelial cells (*p 0.0196*), and fibroblasts (*p 0.009*; Fig. 3a-d). Furthermore, we analyzed the associations of the clusters with clinically relevant variables. Cluster one exclusively comprised malignant prostate tissue and was enriched for significant prostate cancer (*p* < 0.002 likelihood ratio, Fig. 3e). Along that line, more advanced tumor stages, as defined by Gleason scores, were found in cluster one (*p0.012* likelihood ratio, Fig. 3f). Cluster one patients had higher pre-surgical maximum PSA values (Fig. 3g), and cluster one was enriched for higher ISUP grades, i.e., more advanced tumor stages (*p* < 0.0004 likelihood ratio) (Fig. 3h). We leveraged four different machine learning models to non-invasively predict the epigenetic clusters using radiomics analyses. No model could predict the epigenetic clusters non-invasively (ROC AUC ≤ 0.65 for all, Supplementary Fig. 3).

## Discussion

Our data demonstrate that large-scale DNA methylation signatures were associated with relevant pathological and clinical characteristics of patients with prostate cancer. Clinically significant and advanced-stage prostate cancer clustered in a distinct subgroup.

In our study, we identified four main LMCs coalescing into two clusters. LMC-based cluster 1 exclusively contained PCa samples, was composed of more significant PCa samples than cluster 2, harbored cases with higher Gleason scores, and had higher presurgical PSA levels.

No tissue from regions adjacent to tumor tissue (“adjacent benign”) or complete benign BPH tissue (“complete benign”) was allocated to LMC-based cluster 1. The adjacent benign regions, allocated with PCa or complete benign samples, i.e., adjacent benign tissue, did not form its own cluster. A potential reason could be contamination with scattered PCa, but this remains elusive as no CpG site was significantly differentially methylated between complete benign and adjacent benign tissue. Among differentially methylated loci between adjacent benign tissue and PCa, a CpG site associated with the gene *RPL35AP31* was found, which to date has not yet been described in the context of prostate cancer. Mapping to the same genomic location 13q21.33 is the gene dachshund homolog 1 isoform c, which methylation status has recently been shown to correlate with advanced and less radio-sensitive esophageal cancer (Huang et al. 2022). Another CpG site was associated with the gene *ARHGAP42P1 and* not further characterized until now, but listed as enhancer according to the Ensembl database (Martin et al. 2023). The potential relevance of those two genes in prostate cancer needs further investigation in bigger data sets. Currently, neither of the two CpG sites was represented among differentially methylated sites in the Infinium HumanMethylation450 dataset of Geybels et al., which compared 20 PCa samples with matched adjacent benign samples (Geybels et al. 2015). The absence of significant methylation differences between complete and adjacent benign tissue might be interpreted as lacking pre-cancerous or perilesional epigenetic changes in histologically benign-looking prostate tissue or might be biased by our small sample size. However, Zhang et al., who compared genome-wide methylomes of prostate cancer, pre-cancerous lesions, and normal prostatic tissue, showed that average DNA methylation levels dropped in pre-cancerous prostate vs. normal tissue and were elevated in cancer (Zhang et al. 2023). The latter reached statistical significance only when compared with pre-cancerous, not normal tissue (Zhang et al. 2023). We saw significantly higher global methylation levels in PCa versus benign tissue in our data (data not shown).

In our cohort, some PCa samples clustered in LMC-based cluster 2. To explore a potential biological interpretation of LMC cluster formation, we deployed reference-based tumor deconvolution and found cluster 1 to have a higher proportion of cancer cells while showing an overall decreased leukocyte fraction, as indicated by the independent LUMP algorithm. In short, MethylCIBERSORT-inferred proportions of CD4 + effector T-cell and CD56 + NK cells were lower in LMC-based cluster 1, pointing towards a change of the microenvironmental composition in the cluster enriched for malignant samples. Furthermore, the proportions of the main constituents of the tumor stroma, fibroblasts and endothelial cells, were also diminished in LMC-based cluster 1. Despite their role in carcinogenesis and disease progression immune cell infiltrates in PCa were rather poor. PCa is known to rank amongst immune-cold tumors, which is in line with our results. Intratumoral CD8 + T cells have been shown to express PD-1, which hampers anti-tumorigenic activity (Sfanos et al. 2009). Also, advanced tumor stages may be associated with higher T regulatory cell infiltrates (Davidsson et al. 2013; Karpisheh et al. 2021). While we did not see differences in proportions of T reg or CD8 + T cell infiltrates between LMC-based clusters we observed lower estimates for NK cell infiltrates in the cluster 1. This might further corroborate the impact of LMC-based cluster 1, as lower NK cell counts have been shown to be associated with more aggressive tumor stages (Pasero et al. 2015). Within the immune-cold microenvironment of PCa, cancer-associated fibroblasts (CAFs) were shown to be crucial constituents exerting pro-tumorigenic functions. To remodel the extracellular matrix CAFs stimulate mesenchymal cell invasion and angiogenesis, which eventually contributes to tumor invasion (Vickman et al. 2020). Recently, the ratio between tumor and stroma cells was investigated to serve as a biomarker for disease recurrence. Highlighting the difficulties imposed by tumor heterogeneity either a small or extensive stroma amount was found to be associated with earlier tumor recurrence (Ayala et al. 2003) potentially marking the beginning and final events of significant tumor microenvironmental changes. In line with the recently published studies, in our cohort, we found the LMC-based cluster 1, which exclusively contained malignant samples, to have lower numbers of endothelial cells and fibroblasts than the more benign cluster 2. Our results might be interpreted as aggressive early-stage cases in line with the potential temporal heterogeneity hypothesis of Ayala et al. or it might be caused by sample bias in our small feasibility cohort (Ayala et al. 2003). Further, our methylation reference data was not directly fitted to CAFs. As CAFs differ from both normal fibroblasts as well as among each other with regard to receptor expression and secretion products this could bias the analysis and needs to be regarded as a limitation of our study (Franco et al. 2011). Though the MethylCIBERSORT and the LUMP algorithm independently pointed towards lower immune cell amounts in LMC-based cluster 1.

While radiomics revealed prognostic potential in numerous PCa studies, in our cohort, non-invasive radiographic biomarkers could not stratify epigenetic clusters. Potential reasons could be the small sample size of our feasibility study or the limited sensitivity of radiomics compared to epigenetic analyses of immune signatures in a tumor considered immune-cold.

In conclusion, in this feasibility study, we showed that prognostically relevant metrics in prostate cancer were associated with distinct epigenetic clusters. The malignant and more aggressive cluster 1 showed reduced immune cell-related signatures with reduced signatures of CD19 and CD4 cells. Some prostate cancer samples clustered in the more favorable and immunogenic appearing cluster 2. While this study suggests that epigenetic analysis might be able to stratify prostate cancer cases that have the potential to benefit from immunotherapy more than others, the potential therapeutic relevance of this finding needs to be explored in further research.

## Electronic supplementary material

Below is the link to the electronic supplementary material.

Supplementary Material 1

Supplementary Material 2

Supplementary Material 3

Supplementary Material 4

## Data Availability

The datasets used and/or analyzed during the current study are available from the corresponding author on reasonable request.

## Abbreviations

ADC: Apparent diffusion coefficient
BPH: Benign prostatic hyperplasia
DCE: Dynamic contrast-enhanced
DICOM: Digital imaging and communications in medicine
ESUR: European society of urogenital radiology
HoLEP: Holmium laser enucleation of the prostate
ISUP: International society of urological pathology
LUMP: Leucocytes unmethylation for purity
mpMRI: Multiparametric magnetic resonance imaging
PCa: Prostate cancer
PI-RADS: Prostate imaging reporting and data system
RPX: Radical prostatectomy
T2w: T2-weighted
VOI: Volume of interest
