# Supplementary figures and images for: Epigenetic profiling of prostate cancer reveals potential prognostic signatures
Source: J Cancer Res Clin Oncol. 2024 Aug 24;150(8):396. doi: 10.1007/s00432-024-05921-0 (PMC11344710; doi:10.1007/s00432-024-05921-0)

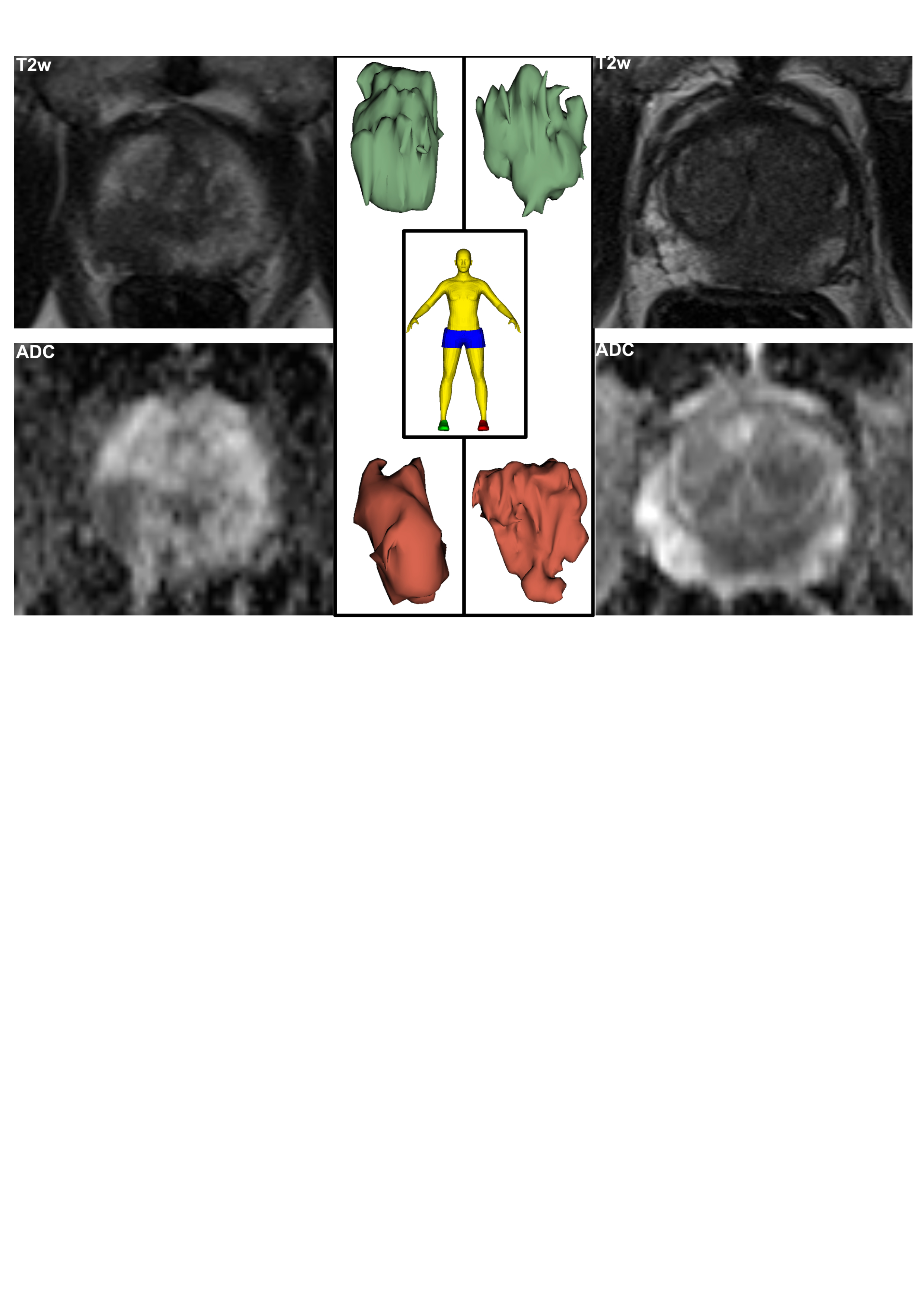

Supplement: Supplementary file 1 — Supplementary Material 1 [file 432_2024_5921_MOESM1_ESM.jpg]

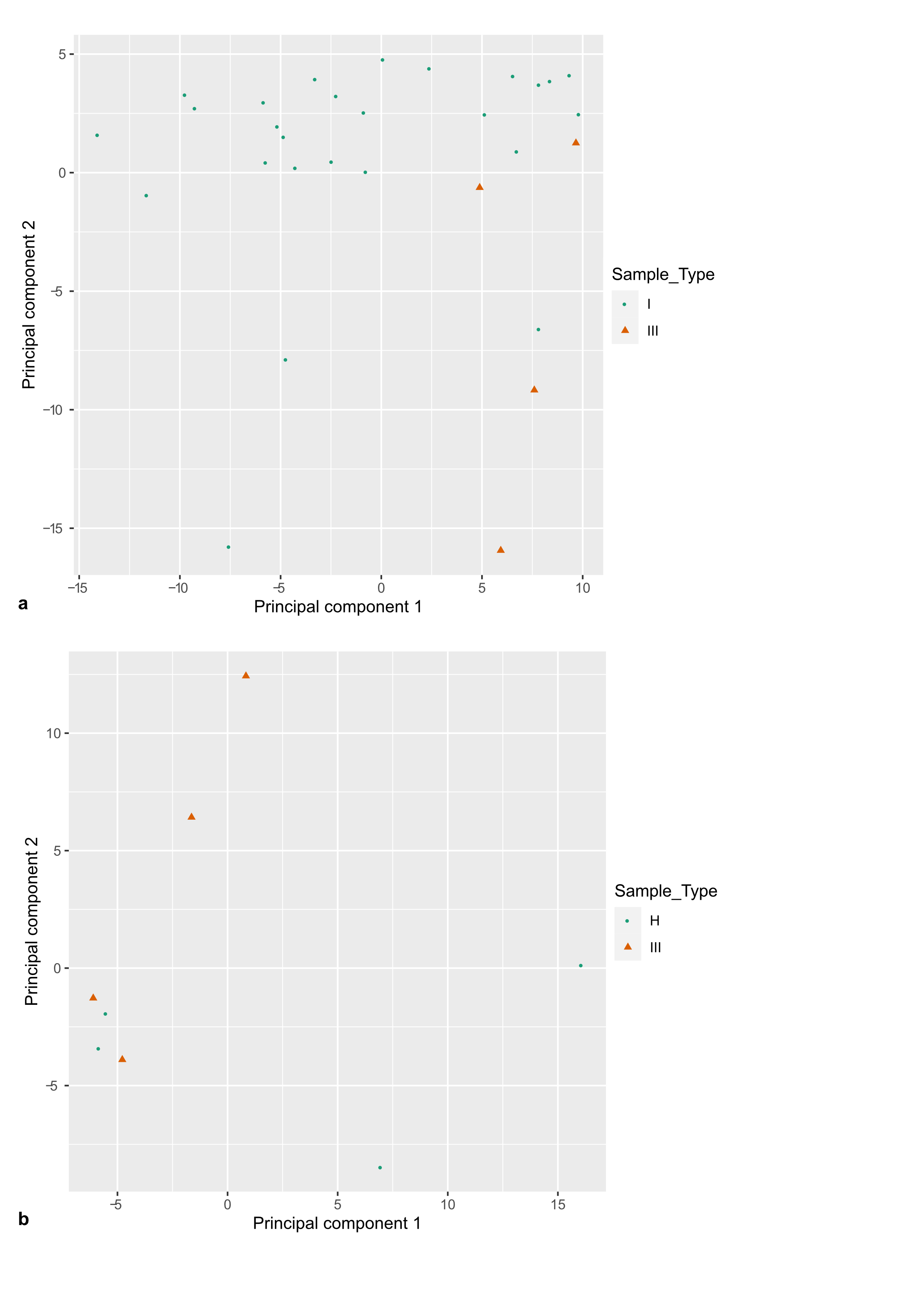

Supplement: Supplementary file 2 — Supplementary Material 2 [file 432_2024_5921_MOESM2_ESM.jpg]

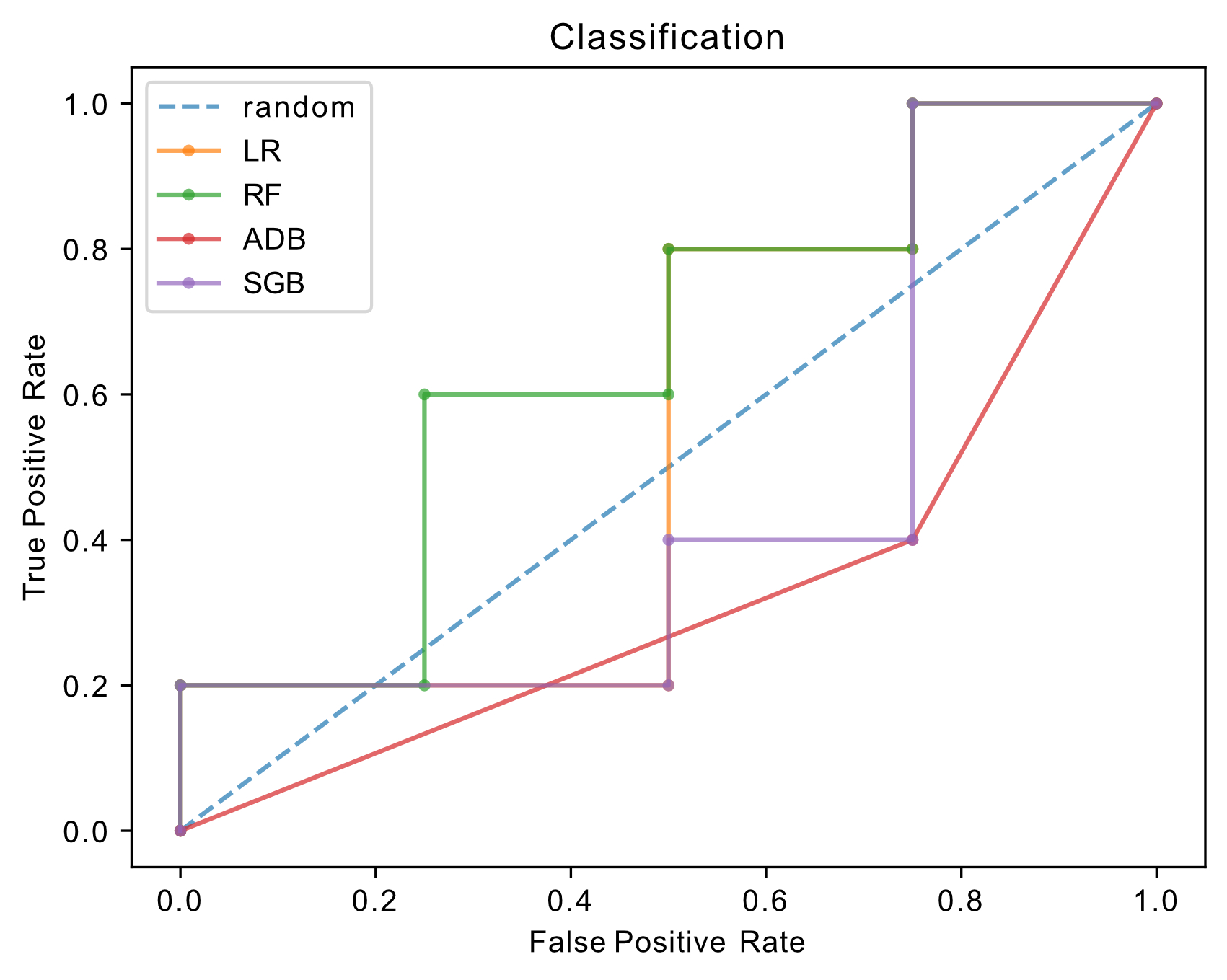

Supplement: Supplementary file 3 — Supplementary Material 3 [file 432_2024_5921_MOESM3_ESM.jpg]
