# Supplementary material for: Epigenetic profiling of prostate cancer reveals potential prognostic signatures
Source: J Cancer Res Clin Oncol. 2024 Aug 24;150(8):396. doi: 10.1007/s00432-024-05921-0 (PMC11344710; doi:10.1007/s00432-024-05921-0)
Supplement: Supplementary file 4 — Supplementary Material 4 [file 432_2024_5921_MOESM4_ESM.docx]

**Supplemental Material**

**Supplementary Figure 1:** *Representative images of prostate cancer segmentation*

Multiparametric MR images of two representative patients to depict the workflow of volume of interest definition (VOI). Left: Patient with ISUP 2, pT3a N0 M0 R0 prostate cancer. Right: Patient with ISUP 5, pT3b N0 M0 R1 prostate cancer. 3D volume rendering is shown for the T2w (top) and ADC (bottom) segmentation for both tumors depicted as seen from frontal view.

**Supplementary Figure 2**: Global differential methylation analysis shows no clear clustering between adjacent benign samples ("III") vs. PCa ("I") and complete benign samples ("H").

a Principal Component Analysis PCa vs. adjacent benign tissue, Percentage of total variance PC1 29.43, PC2 17.77; b Principal Component Analysis complete benign vs. adjacent benign tissue, Percentage of total variance PC1 35.09, PC2 24.19.

**Supplementary Figure 3:** *Radiomics discriminative performance.*

Receiver operating characteristics (ROC). LR logistic regression, RF random forest, ADB AdaBoost, and SGB stochastic gradient boosting

#

# **Supplementary Table 1:** *Multiparametric MRI sequence parameters.*

| Sequence parameter | T2 TSE | ADC | DCE |
| --- | --- | --- | --- |
| TR (ms) | 7737.2 | 3425.0 | 5.08 |
| TE (ms) | 101.9 | 59.2 | 1.8 |
| Averages | 2.3 | 7.8 | 1.0 |
| Flip angle (°) | 160.0 | 90.0 | 13.5 |
| FOV (mm^2^) | 202.0 x 202.0 | 95.3 x 202.5 | 258.2 x 259.0 |
| Matrix (px^2^) | 304.4 x 352.0 | 53.2 x 148.8 | 153.5 x 192.0 |
| Bandwidth (Hz) | 200.0 | 1203.6 | 260.0 |
| Slice thickness (mm) | 3.0 | 3.0 | 3.5 |
| Orientation | transversal | transversal | transversal |
| *b*0_1000 (N)  *b*50_1000 (N) |  | 24  6 |  |

Imaging parameter. ADC, apparent diffusion coefficient; DCE, dynamic contrast enhanced; FOV, field of view; n, absolute number; TE, echo time; TR, repetition time; TSE, turbo-spin-echo.

**Supplementary Table 2:** *Differentially methylated CpG sites between prostate cancer (Pca) and adjacent benign tissue on promoter level.* Differentially methylated sites with FDR adj. p values <.05.

| **id** | **Chromo-some** | **Start** | **End** | **symbol** | **mean.mean**  **Pca** | **mean.mean**  **adjacent benign** | **mean.mean.diff** | **comb.p.adj.fdr** |
| --- | --- | --- | --- | --- | --- | --- | --- | --- |
| ENSG00000227817 | chr2 | 132087931 | 132089930 | ARHGAP42P1 | 0.58109001 | 0.0663441 | 0.51474591 | 0.00044212 |
| ENSG00000239419 | chr7 | 142986208 | 142988207 | RN7SL535P | 0.59203151 | 0.15611887 | 0.43591264 | 0.00867537 |
| ENSG00000233460 | chr13 | 72418613 | 72420612 | RPL35AP31 | 0.35999749 | 0.8331539 | -0.4731564 | 0.00867537 |
| ENSG00000267880 | chr19 | 51377541 | 51379540 | NA | 0.18700917 | 0.67849923 | -0.4914901 | 0.03096385 |
| ENSG00000230832 | chr1 | 146554628 | 146556627 | NA | 0.63459834 | 0.23539007 | 0.39920827 | 0.03720072 |
| ENSG00000231147 | chr2 | 130762273 | 130764272 | ARHGAP42P2 | 0.59555846 | 0.24559412 | 0.34996433 | 0.03720072 |
| ENSG00000213270 | chr12 | 83543610 | 83545609 | RPL6P25 | 0.74621407 | 0.30057745 | 0.44563662 | 0.03720072 |
| ENSG00000211562 | chr8 | 48801119 | 48803118 | NA | 0.4883754 | 0.84963433 | -0.3612589 | 0.03965517 |
| ENSG00000221262 | chr7 | 81917893 | 81919892 | NA | 0.33427836 | 0.75563173 | -0.4213534 | 0.04031611 |
| ENSG00000130513 | chr19 | 18484041 | 18486040 | GDF15 | 0.60112968 | 0.14550814 | 0.45562154 | 0.04031611 |

**Supplementary Table 3:** *Differentially methylated CpG sites between prostate cancer (Pca) and adjacent benign tissue on gene level.* Differentially methylated sites with FDR adj. p values <.05.

| **id** | **Chromosome** | **Start** | **End** | **symbol** | **mean.mean**  **Pca** | **mean.mean**  **adjacent benign** | **mean.mean.diff** | **comb.p.adj.fdr** |
| --- | --- | --- | --- | --- | --- | --- | --- | --- |
| ENSG00000197921 | chr1 | 2460184 | 2461684 | HES5 | 0.55008842 | 0.13892926 | 0.41115916 | 0.01552686 |
| ENSG00000249883 | chr4 | 181652317 | 181680191 | NA | 0.30828491 | 0.75657754 | -0.4482926 | 0.01552686 |
| ENSG00000271788 | chr5 | 43006835 | 43007645 | NA | 0.50795452 | 0.08831988 | 0.41963464 | 0.01552686 |
| ENSG00000124610 | chr6 | 26017260 | 26018040 | HIST1H1A | 0.6037549 | 0.18864552 | 0.41510938 | 0.01552686 |
| ENSG00000226005 | chr10 | 3976711 | 3978005 | NA | 0.65773326 | 0.2146276 | 0.44310566 | 0.01552686 |
| ENSG00000233460 | chr13 | 72420113 | 72420449 | RPL35AP31 | 0.35999749 | 0.8331539 | -0.4731564 | 0.01552686 |
| ENSG00000267880 | chr19 | 51377552 | 51378040 | NA | 0.18700917 | 0.67849923 | -0.4914901 | 0.01552686 |
| ENSG00000250819 | chr4 | 17173380 | 17187682 | NA | 0.35367881 | 0.82193115 | -0.4682523 | 0.02771688 |
| ENSG00000248663 | chr5 | 116751205 | 116881993 | LINC00992 | 0.29429505 | 0.64788127 | -0.3535862 | 0.03283991 |
| ENSG00000269751 | chr19 | 48983104 | 48985571 | NA | 0.66103279 | 0.29136534 | 0.36966745 | 0.03283991 |
| ENSG00000225431 | chr21 | 44019390 | 44035168 | NA | 0.72907322 | 0.33182665 | 0.39724657 | 0.03283991 |
| ENSG00000228251 | chr2 | 113348373 | 113349516 | NA | 0.36931029 | 0.80879671 | -0.4394864 | 0.04037952 |
| ENSG00000254648 | chr11 | 133905727 | 133907002 | NA | 0.66241485 | 0.18193684 | 0.48047801 | 0.04037952 |
| ENSG00000207405 | chr16 | 2012974 | 2013107 | SNORA64 | 0.34292823 | 0.76866827 | -0.42574 | 0.04037952 |
| ENSG00000270164 | chr19 | 42041545 | 42043266 | NA | 0.23959841 | 0.61084147 | -0.3712431 | 0.04117268 |
| ENSG00000248895 | chr17 | 46958484 | 46958678 | NA | 0.33537534 | 0.76274474 | -0.4273694 | 0.04590789 |
| ENSG00000234437 | chr1 | 169411876 | 169429690 | NA | 0.51378012 | 0.16946176 | 0.34431836 | 0.04727533 |
